# Pregnant Women With Multidrug-Resistant/Rifampicin-Resistant Tuberculosis and the All-Oral 6-Month Regimen: Experiences From a Patient Series in South Africa

**DOI:** 10.1093/cid/ciag032

**Published:** 2026-01-22

**Authors:** Marian Loveday, Emma Clarence, Sindisiwe Hlangu, Nalini Singh, Sunitha Chotoo, James C M Brust, Catriona Waitt, Richard Court, Jennifer Furin

**Affiliations:** HIV and Other Infectious Diseases Research Unit, South African Medical Research Council, Durban, South Africa; CAPRISA-MRC HIV-TB Pathogenesis and Treatment Research Unit, Durban, South Africa; HIV and Other Infectious Diseases Research Unit, South African Medical Research Council, Durban, South Africa; HIV and Other Infectious Diseases Research Unit, South African Medical Research Council, Durban, South Africa; King Dinuzulu Hospital Complex, Sydenham, Durban, South Africa; King Dinuzulu Hospital Complex, Sydenham, Durban, South Africa; Divisions of General Internal Medicine and Infectious Diseases, Montefiore Medical Center, Albert Einstein College of Medicine, NewYork, New York, USA; Infectious Diseases Institute, College of Health Sciences, Makerere University, Kampala, Uganda; Department of Women's and Children's Health, University of Liverpool, Liverpool, United Kingdom; Division of Clinical Pharmacology, Department of Medicine, University of Cape Town, Cape Town, South Africa; Department of Global Health and Social Development, Harvard Medical School, Boston, Massachusetts, USA; Division of Infectious Diseases and HIV Medicine, Case Western Reserve University and University Hospitals Cleveland Medical Center, Cleveland, Ohio, USA

**Keywords:** tuberculosis, drug-resistance, all-oral 6-month MDR/RR-TB regimen, outcomes

## Abstract

**Background:**

There is limited experience of the all-oral 6-month regimens containing bedaquiline, delamanid, linezolid, and levofloxacin or clofazimine (BDLLfx/BDLCfz) in pregnant women with multidrug-resistant/rifampicin-resistant tuberculosis (MDR/RR-TB). We report maternal treatment, pregnancy, and infant outcomes to 12 months of age in a cohort of pregnant women treated with these regimens.

**Methods:**

We included pregnant women treated for MDR/RR-TB from September 2023 to January 2025 in KwaZulu-Natal, South Africa, in a prospective observational study. Outcomes were collected through ongoing record reviews. Infant clinical assessments were conducted at 6 weeks, 6 months, and 12 months.

**Results:**

Of 25 pregnant women with MDR/RR-TB, 21 received BDLLfx/BDLCfz; 12 (57%) were diagnosed with human immunodeficiency virus. Although 10 of the 21 (48%) treated women developed anemia, 18 (86%) had favorable treatment outcomes. All 21 infants were born alive, with a median gestational age 39 weeks (interquartile range [IQR], 38–40 weeks) and median birth weight 3160 g (IQR, 2818–3308 g). Three women had unfavorable pregnancy outcomes, with infants born prematurely (2 with low birth weight and 1 who developed respiratory distress syndrome). Of the 18 infants evaluated at 12 months, 10 (56%) had possible or confirmed unfavorable outcomes. Two infants had confirmed congenital anomalies and 3 possible congenital anomalies, but only 1 had first-trimester drug exposure. One infant died, another was diagnosed with MDR/RR-TB and started on treatment, and 3 infants had signs/symptoms of tuberculosis, necessitating referral for care.

**Conclusions:**

These limited data suggest that in pregnant women, the BDLLfx/BDLCfz regimens have improved treatment and pregnancy outcomes compared to prior regimens. However, there is a high prevalence of unfavorable infant outcomes.

## Teaser

In pregnant women with multidrug-resistant/rifampicin-resistant tuberculosis, all-oral 6-month regimens containing bedaquiline, delamanid, linezolid, and levofloxacin/clofazimine have improved treatment and pregnancy outcomes compared to prior regimens, but there is a high prevalence of unfavorable infant outcomes.

In 2022, the World Health Organization (WHO) recommended the use of an all-oral 6-month regimen for people with multidrug-resistant/rifampicin-resistant tuberculosis (MDR/RR-TB) containing bedaquiline (B), pretomanid (Pa), and linezolid (L), with or without moxifloxacin (M) [1]. These regimens, known as the “BPaLM” or BPaL” regimens, were shown to be effective and safe in a large multicountry randomized controlled trial (TB-PRACTECAL) [2, 3]. Pregnant women were excluded from these guidelines as pretomanid is not recommended during pregnancy due to a reproductive toxicity signal observed in animals [4]. Based on preliminary results from the BEAT-Tuberculosis trial, which reported that a 6-month regimen of bedaquiline, delamanid, linezolid, levofloxacin (Lfx), and clofazimine (Cfz) (BDLLfxCfz) was noninferior compared to the 9-month standard of care regimen in South Africa, pregnant women with MDR/RR-TB are now treated with a 6-month delamanid-containing regimen instead: “BDLLfx” for women susceptible to fluoroquinolones or the “BDLCfz” regimen for women with fluoroquinolone resistance [5, 6].

There are limited data describing 6-month treatment regimens for MDR/RR-TB in pregnancy. TB-PRACTECAL included 16 participants who became pregnant during the study [2, 3]; 10 pregnant women were included in BEAT-Tuberculosis, 4 of whom were randomized to the 6-month experimental arm, and treated with a BDLLfxCfz regimen [5]. Given the limited experience using 6-month MDR/RR-TB regimens in pregnant women, we sought to document maternal treatment, pregnancy, and infant outcomes at birth and at 12 months of age in a cohort of pregnant women treated with BDLLfx or BDLCfz.

## METHODS

In this prospective observational cohort study, we enrolled pregnant women ≥18 years of age treated for MDR/RR-TB for ≥14 days at King Dinuzulu Hospital, KwaZulu-Natal, South Africa. Baseline maternal clinical characteristics were captured at enrollment from an initial record review. We conducted bimonthly record reviews to capture maternal response to TB treatment, regimen changes, and treatment outcomes (clinical data are documented monthly by hospital clinicians). At 6 weeks of age, infants were brought to the hospital and data from each infant's *Road to Health* book were extracted to obtain pregnancy outcomes. This book, a patient-held record that is standard of care in South Africa, details the child's medical history, health, growth, and development. Following this, a clinical assessment by a pediatrician was performed and repeated at 6 and 12 months to determine infant outcomes. Maternal TB treatment and pregnancy outcomes for 11 of these women have been reported previously [7]. In this article, in addition to TB treatment and pregnancy outcomes for these 11 women, we also report infant outcomes following clinical assessment up to 12 months.

We defined 3 outcomes—maternal TB treatment outcomes, pregnancy outcomes, and infant outcomes—as either favorable or unfavorable. Maternal TB treatment outcome definitions were based on WHO guidelines, with a favorable outcome (treatment success) defined as either cure or treatment completion, and an unfavorable treatment outcome defined as death, loss to follow-up, or treatment failure [8]. Pregnancy outcomes were classified as favorable if the infant was born alive at term (≥37 weeks of pregnancy) and weighing ≥2500 g [9]. Unfavorable pregnancy outcomes comprised (*i*) fetal or neonatal deaths, which included stillbirths and spontaneous abortions; (*ii*) elective termination of pregnancy at any gestational age; (*iii*) preterm delivery (<37 weeks); and (*iv*) low birth weight (<2500 g). A favorable infant outcome was assigned to an infant who at 12 months of age was thriving (ie, gaining weight and following the normal growth chart trajectory) and achieving developmental milestones, with no confirmed/possible TB or congenital anomalies.

As per South African MDR/RR-TB guidelines, pregnant women were reviewed monthly by hospital clinicians to assess treatment response, document adverse events, and encourage adherence. Weight, visual acuity, electrocardiography, and peripheral neuropathy assessments were performed monthly, in addition to chest radiography and routine laboratory assessments including a full blood count, sputum microscopy, and mycobacterial culture. Adverse events were recorded in the medical records with subsequent drug dose adjustments/discontinuations documented.

At the 6-week infant postpartum visit, key information from the *Road to Health* book was extracted, including management of the infant at birth and whether, in accordance with national guidelines, they were tested for human immunodeficiency virus (HIV-1) and prescribed antiretroviral prophylaxis, screened for TB, and given a BCG vaccine and TB preventive therapy. Clinical assessment by a pediatrician at 6 weeks, 6 months, and 12 months included a physical examination with measurement of infant weight, length, and head circumference; growth curve monitoring; and evaluation of developmental milestones. Infants were screened for signs and symptoms of TB and referred for chest radiography and/or gastric aspirate sampling if indicated. Children with TB disease were classified as (*i*) confirmed TB if they had at least 1 positive culture/TB nucleic acid amplification test from a respiratory specimen, (*ii*) unconfirmed TB if they had suggestive symptoms and chest radiograph consistent with intrathoracic TB disease, and (*iii*) presumptive TB if they had suggestive symptoms or chest radiograph consistent with intrathoracic TB disease [10, 11]. Congenital anomalies were diagnosed based on a clinical examination and if suspected, referred for further investigation and management. Infants were classified as having an unfavorable outcome if, after 12 months, their growth faltered (using the WHO definition of a fall in weight-for-age *z* score of ≥1.0 [12]) or they had not achieved developmental milestones, had a congenital anomaly, were suspected of having TB, or died.

This study was approved by the South African Medical Research Council Ethics Review Committee (EC017-6/2016) and the KwaZulu-Natal Health Research Committee (KZ 202105 013).

## RESULTS

**Figure 1.**
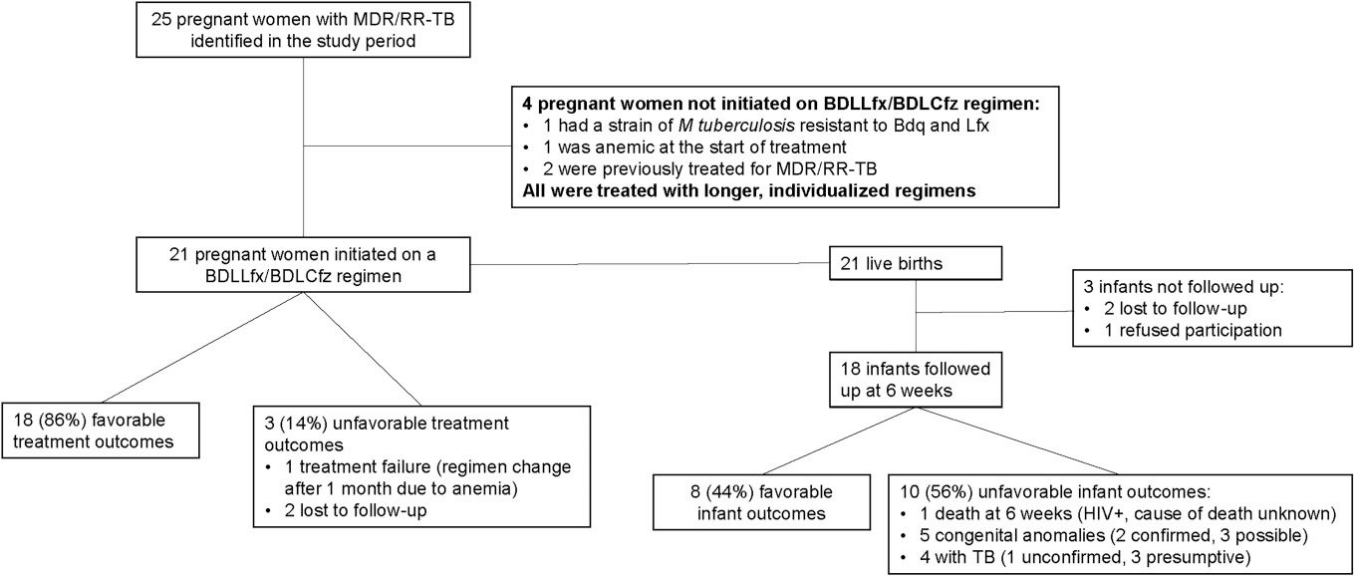
Schema of enrollment and follow-up (1 September 2023–31 August 2025). Abbreviations: BDLCfz, bedaquiline, delamanid, linezolid, and clofazimine; BDLLfx, bedaquiline, delamanid, linezolid, and levofloxacin; Bdq, bedaquiline; HIV, human immunodeficiency virus; Lfx, levofloxacin; *M. tuberculosis*, *Mycobacterium tuberculosis*; MDR/RR-TB, multidrug-resistant/rifampicin-resistant tuberculosis; TB, tuberculosis.

Between 1 September 2023 (when the BDLLfx/BDLCfz regimen was introduced in South Africa) and 31 January 2025, 25 pregnant women were initiated on MDR/RR-TB treatment. Treatment regimens were constructed using South African National TB Programme guidelines including individual patient factors. Four women were not eligible for a 6-month regimen and were initiated on individualized long regimens: (*i*) 1 participant was anemic at the start of treatment (hemoglobin 7.2 g/dL) and initiated on a regimen without linezolid (bedaquiline [Bdq] + delamanid [Dlm] + Lfx + Cfz); (*ii*) another participant was infected with a strain of *Mycobacterium tuberculosis* resistant to both bedaquiline and fluoroquinolones, and therefore treated with Dlm + linezolid [Lzd] + Cfz + terizidone (Trd); and (*iii*) 2 participants had previously been treated for MDR/RR-TB: 1 was treated with Bdq + Lzd + Cfz + Lfx + Trd + para-aminosalicylic acid (Pas), and the other with Bdq + Dlm + Lzd + Trd + Pas + pyrazinamide. Figure 1 details study enrollment and follow-up.

In total, 21 women were treated with a BDLLfx or BDLCfz, 20 were treated with BDLLfx, and 1 with fluoroquinolone resistance received BDLCfz. Linezolid was prescribed for 6 months with discontinuations for adverse events. Nineteen women were already pregnant when they initiated treatment. One became pregnant after initiating treatment with pretomanid (BPaLLfx regimen), which was subsequently replaced with delamanid (total infant exposure to pretomanid was 3 months). Another woman was treated with BDLLfx for 1 month, and after developing anemia, changed to a linezolid-sparing long regimen. Gestational age at the start of treatment ranged from 8 to 37 weeks and the median gestational age at treatment initiation was 24 weeks (interquartile range [IQR], 19.5–30.0 weeks) (Table 1). Three women (14%) were initiated on MDR/RR-TB treatment during the first trimester, 12 (57%) during the second trimester, and 6 (29%) in the third trimester.

**Table 1.** Baseline Clinical Characteristics of Pregnant Women With Multidrug-Resistant/Rifampicin-Resistant Tuberculosis Who Were Treated With the 6-Month BDLLfx/BDLCfz Regimen

Characteristics	N = 21^a^
Clinical characteristics	
Age, y, mean ± SD	28.4 ± 7.5
Hemoglobin, g/dL, mean ± SD	11.1 ± 2.5
BMI, kg/m^2^, mean ± SD	28.1 ± 5.0
BMI category, kg/m^2^ (n = 19)	
Underweight (<18.5)	0
Normal (18.5–24.9)	3 (16)
Overweight (25–29.9)	9 (47)
Obese (≥30.0)	7 (37)
TB characteristics	
Smear positive at treatment initiation (n = 19)	7 (37)
Culture positive at treatment initiation (n = 20)	9 (45)
Previous TB or MDR/RR-TB (n = 19)	5 (26)
Site of TB: pulmonary	21 (100)
Chest radiograph (n = 18)	
Extensive disease on chest radiograph^b^	6 (33)
Minimal disease	11 (61)
Normal	1 (6)
Resistance pattern	
RR-/Rif-mono/MDR-TB	19 (90)
MDR-TB + fluoroquinolone resistance	1 (5)
INH-monoresistant TB	1 (5)
HIV characteristics	
Diagnosed with HIV	12 (57)
Diagnosed with HIV on ART before MDR/RR-TB treatment started (n = 12)	11 (92)
Baseline CD4 count, cells/μL, median (IQR)	844 (577.5–888)
Baseline HIV viral load (n = 12)	
Undetectable (<40 copies/mL)	10 (83)
Pregnancy characteristics	
Pregnant before MDR/RR-TB treatment started	19 (90)
Gestational age at treatment start, wk, median (IQR)	24 (19.5–30.0)
Trimester during which treatment started	
Trimester 1	3 (14)
Trimester 2	12 (57)
Trimester 3	6 (29)

Data are presented as No. (%) unless otherwise indicated.

Abbreviations: ART, antiretroviral therapy; BDLCfz, bedaquiline, delamanid, linezolid, and clofazimine; BDLLfx, bedaquiline, delamanid, linezolid, and levofloxacin; BMI, body mass index; HIV, human immunodeficiency virus; INH, isoniazid; IQR, interquartile range; MDR/RR-TB, multidrug-resistant/rifampicin-resistant TB; Rif-mono TB, rifampicin-monoresistant tuberculosis; SD, standard deviation; TB, tuberculosis.

^a^Variable N, as specified, excluding missing data.

^b^Bilateral lung disease and/or cavities.

Twelve (57%) of the women in the cohort were diagnosed with HIV. All were treated with the WHO-recommended first-line antiretroviral therapy (ART) regimen with tenofovir disoproxil fumarate, lamivudine, and dolutegravir.

### Maternal Treatment and Pregnancy Outcomes

Of the 21 women treated with BDLLfx/BDLCfz, favorable treatment outcomes were reported in 18 (86%) women (Table 2). All women delivered a live infant. Eighteen of the 21 (86%) had favorable pregnancy outcomes. Of the 3 unfavorable pregnancy outcomes, 1 infant was born at 34 weeks of gestational age weighing 1880 g. Following an emergency cesarean delivery, this infant developed respiratory distress syndrome and was hospitalized for 9 days, requiring noninvasive respiratory support for 2 days. At the 6-week, 6-month, and 12-month assessments, the infant was gaining weight and had achieved appropriate developmental milestones. Two infants were born prematurely, 1 at 34 weeks of gestational age weighing 2350 g, the second at 36 weeks weighing 3000 g.

**Table 2.** Maternal Treatment, Pregnancy, and Infant Outcomes for Pregnant Women Treated With the 6-Month BDLLfx/BDLCfz Regimen

Outcome	First-Trimester Drug Exposure (n = 2)	Second-Trimester Drug Exposure (n = 12)	Third-Trimester Drug Exposure (n = 7)	Total (N = 21)
Maternal treatment outcomes				
Favorable treatment outcomes	2	11	5	18 (86%)
Cure	1	5	3	9
Treatment completed	1	6	2	9
Unfavorable treatment outcomes	0	1	2	3 (14%)
LTFU	0	0	2	2
Died	0	0	0	0
Treatment failed	0	1	0	1
Pregnancy outcomes				
Live births	2	12	7	21 (100%)
Gestational age at delivery, wk, median (IQR)				39 (38–40)
Birth weight, g, median (IQR)				3160 (2818–3308)
Favorable pregnancy outcomes^a^	2	10	6	18 (86%)
Unfavorable pregnancy outcomes^b^	0	2	1	3 (14%)
Infant outcomes at 12 mo				
No infant outcomes: LTFU/refused participation	1	1	1	3
Infant outcomes	2	10	6	18
Favorable infant outcomes^c^	1	3	4	8 (44%)
Confirmed or possible unfavorable infant outcomes^d^	1	7	2	10 (56%)

Abbreviations: BDLCfz, bedaquiline, delamanid, linezolid, and clofazimine; BDLLfx, bedaquiline, delamanid, linezolid, and levofloxacin; IQR, interquartile range; LTFU, lost to follow-up; TB, tuberculosis.

^a^A favorable pregnancy outcome is defined as an infant born at term (≥37 weeks) with a birthweight ≥2500 g.

^b^An unfavorable pregnancy outcome is defined as an infant born preterm (<37 weeks) or with a low birth weight (<2500 g).

^c^A favorable infant outcome is defined as an infant who at 12 months is thriving (ie, gaining weight and following the normal trajectory according to the growth chart) and developing normally, with no possible or confirmed TB or congenital anomaly.

^d^Confirmed and possible unfavorable infant outcomes are detailed in Table 4.

### Maternal Adverse Effects

The most frequent adverse effect was anemia (n = 10 [48%]). The dose of linezolid was never modified but discontinued in 4 women. One of these women developed anemia after 2 weeks; linezolid was discontinued, she was subsequently changed to an individualized long regimen (Bdq + Dlm + Cfz + Trd + Pas), and, in line with WHO guidelines, was classified as having failed treatment. Three other women developed anemia after 12 weeks of treatment, requiring discontinuation of linezolid. In 1 of the 3, linezolid was later restarted. Linezolid was not reinitiated in the remaining 2 women who had responded well to treatment and culture converted; treatment for 1 of these women was extended for an additional 3 months.

Linezolid was stopped in another woman due to a decline in visual acuity. Four women experienced vomiting after the initiation of treatment, which subsided. One of these women recommenced vomiting after becoming pregnant during MDR/RR-TB treatment. Although there were no reports of diabetes mellitus or gestational diabetes, 2 women experienced symptomatic episodes of hypoglycemia. All adverse events reported are documented in Supplementary Table 1.

### Infant Outcomes

Eighteen of the 21 infants were assessed at 12 months. Two women and their infants did not attend the 6-week clinical assessment, and the father of the third infant refused further participation in the study. Thirteen (72%) of the assessed infants were breastfed. Eight (44%) infants had favorable outcomes (Table 2). However, 10 infants (56%), 6 of whom were HIV-exposed, had unfavorable outcomes (Table 3). Two infants were born with confirmed congenital anomalies: the first with a cyanotic congenital cardiac lesion that included pulmonary stenosis, a small atrial septal defect, and possible patent foramen ovale, and the second with an umbilical hernia. Three infants had possible congenital anomalies: (*i*) macrocephaly with deformational plagiocephaly and facial asymmetry, (*ii*) Beckwith–Wiedemann syndrome (macrosomia and umbilical hernia) [13], and (*iii*) macrocephaly. Only 1 of the 5 infants (the infant with macrocephaly with deformational plagiocephaly) had MDR/RR-TB drug exposure during the first trimester. One infant, who was diagnosed with HIV, died at 6 weeks; the cause of death is unknown as the delivery and infant records were not available. The remaining 4 infants had signs and symptoms of TB. In addition to signs and symptoms, 1 infant with chest radiograph changes suggestive of TB, but no laboratory confirmation, was considered to have unconfirmed TB and was initiated on the same MDR/RR-TB treatment regimen as the mother. Three infants, classified as presumptive TB as they had signs and symptoms of TB, were referred for further investigations and follow-up, but did not attend. Clinical details and subsequent follow-up information are tabulated in Table 4. The skin color of the infant born to the women treated with a clofazimine-containing regimen was considered normal by both mother and clinician. Although the mother's adherence was suboptimal, this was not related to clofazimine.

**Table 3.** Confirmed and Possible Unfavorable Infant Outcomes in Pregnant Women Treated With the 6-Month BDLLfx/BDLCfz Regimen

Age, y	Maternal HIV Status	CD4 Count at TB Treatment Initiation, Cells/μL	Viral Load at Treatment Initiation, Copies/mL	Trimester and WGA at MDR/RR-TB Treatment Initiation	Unfavorable Infant Outcomes	Presumptive TB
29	Positive	42	80	Third trimester; 31 WGA	Infant death at 6 wk of age	Insufficient clinical information
29	Positive	566	<40	Second trimester; 13 WGA	Confirmed congenital anomaly: cyanotic congenital cardiac lesion (pulmonary stenosis/small atrial septal defect/possible patent foramen ovale)	No
34	Positive	324	<40	Second trimester; 13 WGA	Confirmed congenital anomaly: umbilical hernia	No
32	Positive	892	<40	First trimester; 8 WGA	Possible congenital anomaly: macrocephaly	No
31	Positive	760	<40	Second trimester; 22 WGA	Possible congenital anomaly^a^: clinical features of Beckwith–Wiedemann spectrum [13]	No
25	Negative	NA	NA	Second trimester; 26 WGA	Possible congenital anomaly: macrocephaly	No
24	Positive	12	164 063	Third trimester; 31 WGA	Unconfirmed perinatal TB^b^	Yes
21	Negative	NA	NA	Second trimester; 19 WGA	Presumptive TB^b^	Yes
40	Negative	NA	NA	Second trimester; 23 WGA	Presumptive TB^b^	Yes
24	Negative	NA	NA	Second trimester; 27 WGA	Presumptive TB^b^	Yes

Abbreviations: BDLCfz, bedaquiline, delamanid, linezolid, and clofazimine; BDLLfx, bedaquiline, delamanid, linezolid, and levofloxacin; HIV, human immunodeficiency virus; MDR/RR-TB, multidrug-resistant/rifampicin-resistant tuberculosis; NA, not applicable; TB, tuberculosis; WGA, weeks of gestational age.

^a^The infant was referred to a pediatric geneticist, but relevant testing not available in the public health service.

^b^Infant TB classified according to an adapted version of Graham and colleagues' 2015 clinical case definitions for TB in children [10] as described in the Methods.

**Table 4.** Clinical Details and Follow-up Information on Infants With Possible or Confirmed Unfavorable Outcomes

Unfavorable Infant Outcome	6-Week Review	6-Month Review	12-Month Review	Presumptive TB
Infant death at 6 wk of age: Mother suffered pregnancy complications including pregnancy-induced hypertension and preeclampsia, hypoglycemia, and severe anemia, requiring a blood transfusion	The infant, who was born with HIV, died at home at 6 wk old, prior to clinical review. The cause of death unknown as delivery and infant records are unavailable, but the mother reported that the infant had breathing difficulties. The mother was LTFU after her infant died, after 3 mo of MDR/RR-TB treatment.	NA	NA	Insufficient clinical information
Cyanotic congenital cardiac lesion (confirmed congenital anomaly): pulmonary stenosis/small atrial septal defect/possible patent foramen ovale	Symptoms: Asymptomatic	Symptoms: Not consistent with TB; single reported episode of blue lips	Symptoms: Asymptomatic	No
	Examination: Abnormal, not consistent with TB; pansystolic cardiac murmur	Examination: Abnormal, not consistent with TB; peripheral cyanosis, pansystolic cardiac murmur, and single second heart sound	Examination: Abnormal, not consistent with TB; findings unchanged as cardiac anomaly persists	
Umbilical hernia (confirmed congenital anomaly)	Symptoms: Asymptomatic	Symptoms: Asymptomatic	Symptoms: Asymptomatic	No
	Examination: Abnormal, not consistent with TB; large reducible umbilical hernia	Examination: Abnormal, not consistent with TB; findings unchanged as umbilical hernia persists	Examination: Abnormal, not consistent with TB; findings unchanged as umbilical hernia persists	
Macrocephaly (possible congenital anomaly)^a^	Symptoms: Asymptomatic	Symptoms: Asymptomatic	Symptoms: Asymptomatic	No
	Examination: Abnormal, not consistent with TB; deformational plagiocephaly with normal head circumference (≥1.6 SD), jittery with upper limb hypertonia; referred to local hospital but did not attend	Examination: Abnormal, not consistent with TB; deformational plagiocephaly with ear shift and facial asymmetry, macrocephaly (> +2 SD); rest of neurological examination normal with normal neurodevelopment; referred to local hospital but did not attend	Examination: Abnormal, not consistent with TB; deformational plagiocephaly with ear shift and facial asymmetry (improving), macrocephaly (> +2 SD, but improving; rest of neurological examination normal with normal neurodevelopment	
Clinical features of Beckwith–Wiedemann spectrum [13] (possible congenital anomaly): (*i*) birthweight >2 SDs above the mean, and (*ii*) umbilical hernia. No other phenotypic features of classical Beckwith–Wiedemann syndrome. Anthropometry at birth: weight for age and head circumference > +2.5 SDs and length for age > +3 SDs.	Symptoms: Asymptomatic	Symptoms: Asymptomatic	Visit pending	No
	Examination: Abnormal, not consistent with TB; macrosomia, reducible umbilical hernia; referred for pediatric genetic testing, but relevant testing not available in the public health service	Examination: Abnormal, not consistent with TB; macrosomia, reducible umbilical hernia		
Macrocephaly (possible congenital anomaly)	Symptoms: Asymptomatic	Symptoms: Asymptomatic	Symptoms: Asymptomatic	No
	Examination: Normal (head circumference ≥1.6 SD)	Examination: Abnormal examination, not consistent with TB; macrocephaly (>+3 SD); neurological examination unremarkable; rest of examination normal. Referred for further assessment: considered to be familial; no investigations undertaken.	Examination: Abnormal examination, not consistent with TB; macrocephaly (> +3 SD); rest of examination normal	
Unconfirmed perinatal TB^b^: 3 wk after delivery, the infant became ill and was admitted with pneumonia	Symptoms: Consistent with TB; respiratory distress	Symptoms: Asymptomatic	Visit pending	Yes
	Examination: Abnormal, consistent with TB; crepitations on chest auscultation; CXR showed bilateral pneumonic changes; treated with oxygen and IV antibiotics. Although NAAT and culture were negative, he was admitted and started on empiric MDR/RR-TB treatment, the same regimen as his mother.	Examination: Hepatosplenomegaly; referred for further investigation		
Presumptive TB^b^	Symptoms: Asymptomatic	Symptoms: Asymptomatic	LTFU	Yes
	Examination: Abnormal, consistent with TB; growth faltering; CXR suspicious for TB; referred for gastric washings but did not attend.	Examination: Abnormal, consistent with TB; moderate acute malnutrition; repeat CXR normal; referred for further investigation and management		
Presumptive TB^b^	Symptoms: Asymptomatic	Symptoms: Asymptomatic	LTFU	Yes
	Examination: Normal but CXR showed bronchiectasis; referred to local hospital but did not attend	Examination: Abnormal, consistent with TB; the infant had generalized lymphadenopathy, hepatomegaly, and poor weight gain, although the CXR had improved. The infant was referred to the local hospital again.		
Presumptive TB^b^	Symptoms: Asymptomatic	Symptoms: Abnormal, consistent with TB; cough and persistent fever	Symptoms: Abnormal, not consistent with TB; cough and nasal congestion, afebrile	Yes
	Examination: Normal			
		Examination: Abnormal, consistent with TB; bilateral wheezes on chest auscultation, hepatomegaly; normal CXR; referred for TB workup	Examination: Abnormal, consistent with TB; bilateral wheezes and transmitted sounds (diagnosed with viral bronchiolitis); CXR normal; growing well	

Abbreviations: CXR, chest X-ray; HIV, human immunodeficiency virus; IV, intravenous; LTFU, lost to follow-up; MDR/RR-TB, multidrug-resistant/rifampicin-resistant tuberculosis; NA, not applicable; NAAT, nucleic acid amplification test; SD, standard deviation; TB, tuberculosis.

^a^Unable to definitively conclude whether macrocephaly is congenital or secondary as, despite referral, no further investigations were undertaken.

^b^Infant TB classified according to an adapted version of Graham and colleagues' 2015 clinical case definitions for TB [10] as described in the Methods.

## DISCUSSION

In this prospective study of 21 women treated for MDR/RR-TB with an all-oral 6-month BDLLfx/BDLCfz regimen in South Africa, 18 (86%) had favorable treatment outcomes. In 2 recent MDR/RR-TB treatment randomized controlled trials that included novel and repurposed drugs, women who became pregnant during the trials were not excluded, and their treatment and pregnancy outcomes were subsequently reported. The 16 pregnant women included in the TB-PRACTECAL study were all treated with bedaquiline and linezolid, of whom 4 also received pretomanid; all 16 had favorable treatment outcomes [14]. In BEAT-Tuberculosis, 4 of the 10 pregnant women included in the study were in the experimental arm and received BDLLfxCfz; 3 had favorable treatment outcomes, but 1 woman had a recurrence of TB 7 months after completing treatment [5]. The STEM-TB study, a prospective cohort study in Kazakhstan, investigated the safety and efficacy of 3 different, all-oral 9-month regimens for MDR/RR-TB [15]. Of 43 pregnant women included in the study, 42 (98%) had successful treatment outcomes [16]. Fewer participants in our study (18 [86%]) had favorable maternal treatment outcomes than those reported in the TB-PRACTECAL and STEM-TB trials, but this is unsurprising given the operational setting of our study. The proportion with favorable outcomes in this current cohort is an improvement from the 67% and 61% we reported in our 2013–2017 and 2018–2024 cohorts, respectively [7, 17]. All 108 women in the first cohort received an 18- to 24-month regimen; 58 received a bedaquiline-containing regimen and 50 women a regimen that included an injectable. In the second cohort, 59 women received an all-oral 9- to 11-month bedaquiline-containing regimen and 11 women the new BDLLfx/BDLCfz regimen. The improvement in the current cohort could in part be explained by the lower proportion of women with HIV (57%), compared to 80% and 76% in our 2013–2017 and 2018–2024 cohorts, respectively [7, 17]. However, the new BDLLfx/BDLCfz regimen may also have contributed to improved treatment outcomes. In 2024, KwaZulu-Natal province reported that 70% of the 1660 patients started on the BPaLLfx/BPaL regimen had favorable outcomes [18].

All 21 women in our cohort delivered a live infant, and 18 (86%) of the pregnancy outcomes were favorable. The pregnancy outcomes we report compare favorably with those of the TB-PRACTECAL trial, endTB observational study, and STEM-TB study [14, 16]. In TB-PRACTECAL, pregnancy outcomes were known for 14 participants; besides 4 abortions (2 elective and 2 spontaneous), 10 live births were reported. Birth weight was reported for 8 neonates, in whom it was normal [14]. In the endTB observational and STEM-TB studies, several pregnancies were terminated early, but of the 31 that continued, there were 26 live births and 2 miscarriages. Birth weight and gestational age were known for 22 infants; 15 (68%) had normal birth weights and 13 (59%) were born at term [16]. In BEAT-Tuberculosis, of the 4 pregnant women treated with a 6-month BDLLfxCfz regimen, all delivered live infants at term [5]. In our 2013–2018 cohort, only 52% of the women had favorable pregnancy outcomes as over a third of infants (35%) were low birth weight, with low birth weight reported in more neonates exposed to bedaquiline compared to neonates not exposed (45% vs 26%; *P* = .034) [17]. In our 2018–2024 cohort, 66% of the pregnancy outcomes reported were favorable and bedaquiline exposure was no longer associated with low birth weight [7]. For the whole cohort (2013–2025), bedaquiline exposure was found not to be associated with low birth weight; 38 of 124 (30.6%) of the infants exposed to bedaquiline had low birth weight, compared to 9 of 48 (18.9%) of the infants not exposed to bedaquiline (*P* = .13).

Anemia, the most frequent maternal adverse effect, was reported in 48% of our study cohort. This is unsurprising in a cohort of pregnant women being treated with a linezolid-containing regimen, as a pregnancy-related effect on hemoglobin concentration (the increase in blood plasma volume in pregnancy exceeds the increase in red cell mass) may have contributed to the high frequency of anemia that we observed [19]. The association of anemia with linezolid has been reported in several studies, including 2 systematic reviews and a meta-analysis, neither of which mentions pregnancy [20, 21].

One strength of our study was the prospective follow-up of all infants for 12 months. It is concerning however, that almost half of the infants were classified as having unfavorable outcomes. We observed 2 confirmed and 3 possible congenital anomalies, although only 1 of these infants had MDR/RR-TB drug exposure during the first trimester. Four of the 5 infants were exposed to HIV and ART perinatally, suggesting a possible compound effect between HIV, ART, MDR/RR-TB, and anti-TB therapy, which should be explored in future cohorts. We did not assess for other potential risk factors for congenital anomalies. In a study of >10 000 live births in our setting, congenital anomalies were reported in 0.5% of ART-exposed infants [22]. A systematic review and meta-analysis of >53 000 infants reported a 10% higher risk of congenital anomalies in infants exposed to ART, a risk that increased in infants exposed to integrase inhibitors [23]. All infants in our study were exposed to the integrase inhibitor dolutegravir in utero. Although initial reports indicated a possible safety signal for neural tube defects associated with dolutegravir exposure, with additional exposure data, the signal has declined [24, 25]. In South African children, the incidence of congenital heart disease is 8 per 1000 live births and the prevalence of umbilical hernia is 61.8% [26, 27].

Our rates of unfavorable infant outcomes are higher than those reported in a small cohort of 6 children exposed to second-line TB drugs in Peru [28]. However, no infants in this study were exposed to HIV/ART and follow-up assessments were performed >3 years after second-line TB drug exposure. Although the mother of the 1 infant exposed to pretomanid assured us via telephone that her infant was well, she did not bring her infant to the 6-week clinical assessment.

Our study has important limitations. First, considering this was an observational study conducted in the public sector using routinely collected data, adverse events may have been underreported, particularly if they were minor and did not require treatment modification. Second, as infant assessments were largely clinical and limited to 3 time points, our ability to infer long-term effects or developmental trajectories was limited. Third, although our study was performed at an operational center and has relevance for similar settings, the single-site study design limits generalizability.

## CONCLUSIONS

Our data, although limited, suggest that in pregnant and postpartum women, the BDLLfx/BDLCfz regimens have improved treatment and pregnancy outcomes compared to historic cohorts. However, the high rates of confirmed or possible congenital anomalies we observed is worrisome and requires further exploration. Our data do not allow a causality assessment, but we note that only 1 of these infants had MDR/RR-TB drug exposure during the first trimester and 4 of these infants were both HIV and MDR/RR-TB exposed. Given the risks associated with some other second-line anti-TB drugs and the clear benefits of shorter treatment regimens, our findings should not infer limited use of BDLLfx/BDLCfz regimens during pregnancy. Rather, our data should prompt close monitoring, clinical follow-up, and support of all infants exposed to anti-TB drugs in utero for a period of 24 months. Furthermore, robust pharmacovigilance in pregnant and breastfeeding women treated for MDR/RR-TB is required to monitor potential fetal effects related to antenatal exposure to novel and repurposed anti-TB-drugs for which there are few data. Pregnancy/infant registries and reciprocal data collection in TB and Maternal and Child Health programs are critical to accumulate sufficient data to assess the risk of rare outcomes including congenital anomalies.

## Supplementary Material

ciag032_Supplementary_Data

## Supplementary Data

Supplementary materials are available at *Clinical Infectious Diseases* online. Consisting of data provided by the authors to benefit the reader, the posted materials are not copyedited and are the sole responsibility of the authors, so questions or comments should be addressed to the corresponding author.
