# Supplementary material for: Pregnant Women With Multidrug-Resistant/Rifampicin-Resistant Tuberculosis and the All-Oral 6-Month Regimen: Experiences From a Patient Series in South Africa
Source: Clin Infect Dis. 2026 Jan 22;82(5):e1063–71. doi: 10.1093/cid/ciag032 (PMC13189674; doi:10.1093/cid/ciag032)
Supplement: ciag032_Supplementary_Data [file ciag032_supplementary_data.docx]

**SUPPLEMENTARY MATERIAL**

**Table 1: Adverse events in pregnant women treated with the 6-month BDLLfx/BDLCfz regimen**

| Adverse events | n=21 |
| --- | --- |
| Anaemia | n=10 (48%) |
| Haemoglobin dropped but linezolid not stopped | 6 |
| Haemoglobin dropped and linezolid stopped | 2 |
| Haemoglobin dropped, linezolid stopped and restarted | 1 |
| Haemoglobin dropped, regimen changed, and linezolid excluded | 1 |
| Gastrointestinal symptoms: Vomiting after taking treatment | n =4 (19%) |
| Vomiting after treatment initiation, which subsided with time | 2 |
| Vomiting after treatment initiation, treatment stopped and   restarted 2 weeks later | 1 |
| Vomiting after treatment initiation which subsided. Vomiting  recommenced when participant fell pregnant | 1 |
| Electrolyte imbalances | n =1 (5%) |
| Hypocalcaemia | 1 |
| Neurological disturbances | n=6 (29%) |
| Vision impairment | 2 |
| Dizziness | 2 |
| Memory loss, headaches, fainting | 1 |
| Painful feet | 1 |
| Skin rash | n=1 (5%) |
| Pregnancy-induced | n =3 (14%) |
| Hypertension | 1 |
| Hypoglycaemia | 2 |

Abbreviations: BDLLfx, Bedaquiline, delamanid, linezolid and levofloxacin; BDLCfz, Bedaquiline, delamanid, linezolid and clofazamine
